# Care management for severely frail older adults in acute & community settings: A qualitative perspective from nurses

**DOI:** 10.1093/geroni/igaf122.2003

**Published:** 2025-12-31

**Authors:** Betsy Seah, Xuan Min Ng, Nandhini Nadarajah, Myint Myint Than

**Affiliations:** National University of Singapore, Singapore, Singapore; National University of Singapore, Singapore, Singapore; National University of Singapore, Singapore, Singapore; Alexander Hospital, Singapore, Singapore

## Abstract

**Background:**

Despite nurses playing a crucial role in the continuity of care for severely frail older adults from hospital to home, there is limited understanding towards their care delivery across these settings.

**Aim:**

To explore the factors influencing nurses’ care management for severely frail older adults in acute and community care settings.

**Methods:**

In this descriptive qualitative study, 18 inpatient and community care nurses who cared for older adults with a clinical frailty score ≥7 were purposively selected from a hospital in Singapore. Individual in-depth interviews were conducted, audio-taped and transcribed verbatim. Data was analysed using reflexive thematic analysis.

**Results:**

The three identified themes are ‘addressing intricacies of care’, ‘navigating care in the community’, and ‘engaging caregivers in care’. Other than providing holistic, dignified care and working in multidisciplinary teams, having specialised training and cultural competency were vital in managing intricate care needs. Despite existing workstreams to integrate health and social care, participants faced financial and structural barriers and experienced limited resources when navigating care. Participants highlighted the importance and challenges of engaging caregivers in caring for the severely frail at home and provided insights on rapport-building and communication.

**Conclusion:**

This study provided insights and strategies to improve care delivery for severely frail older adults. The findings revealed the need to normalise serious illness conversations, increase interdisciplinary training, develop care-specific guidelines for severe frailty, develop cultural competence among internationally educated nurses, enhance community nurses’ competencies, improve efficiency and flexibility of community care services, and develop rapport-building strategies for caregivers.

